# The Impact of Verb Speed on the Processing of Goal-Oriented Visual Scenes

**DOI:** 10.5334/joc.510

**Published:** 2026-08-03

**Authors:** Piia Taremaa, Nele Ots, Ann Veismann, Laura J. Speed

**Affiliations:** 1 University of Tartu, EE; 2 University of Stuttgart, DE; 3 Radboud University, NL

**Keywords:** mental simulation, eye-tracking, verb semantics, speed, motion events, Estonian

## Abstract

Research on mental simulation in language comprehension has yielded mixed findings, with some studies reporting evidence for sensorimotor simulation during language processing and others not. The current study is a conceptual cross-linguistic replication of two eye-tracking experiments on the online processing of speed-induced language originally conducted in English. Participants heard sentences containing a fast- or a slow-motion verb in Estonian (e.g., *Laps vudis/tatsas puu juurde* ‘The child scurried/toddled to the tree.’), while their eye-movements to a corresponding image with an Agent, Path, and Goal were recorded. We were only partly able to find indications of mental simulation triggered by verb semantics. When sentences were longer and more complex (including a Path phrase such as ‘along the path’), the effect of verb speed (fast vs. slow) on eye-movements varied as a function of scene type (with or without a distractor). Time-course analysis suggested attention allocation differences with earlier looks to the Goal in response to slow compared to fast sentences, but only when the sentences were longer and the scenes had no distractor. These mixed findings make it difficult to formulate strong conclusions about mental simulation of speed. If mental simulation of speed exists, it appears to be less consistently observed in visual-world studies than mental simulation of other information researched in the literature, such as colour. The study highlights the importance of addressing mental simulation in a diverse set of studies and of extending them beyond Indo-European languages.

## 1. Introduction

Embodied approaches to language propose that language comprehension involves the reactivation of sensory and motor systems engaged in actual experience. For example, when hearing the word *kick*, action-related regions of the brain activate (Hauk et al., 2004; Tettamanti et al., 2005). This process is known as *mental simulation* (e.g., Zwaan, 2003), and it has been an important line of inquiry in cognitive language research for decades. An accumulating body of research suggests that language processing is closely linked to sensorimotor experiences (Bechtold et al., 2023; e.g., Fischer & Zwaan, 2008; Pulvermüller & Fadiga, 2010). At the same time, studies of mental simulation do not always replicate, highlighting the complexity and context-dependency of mental simulation (Ibáñez et al., 2023), the challenges in accurately capturing mental simulation through experimental designs (Körner et al., 2023), and potentially supporting weaker or unembodied accounts of semantic processing (e.g., Collins & Loftus, 1975; Mahon & Caramazza, 2008; see also Meteyard et al. 2012 for a continuum of embodiment).

This has underscored the need for converging evidence, such as 1) multi-methodological approaches, where the same phenomenon is investigated from diverse perspectives and with varying types of experiments; 2) replication studies, extending to languages beyond English and other Indo-European languages; and 3) open science and pre-registration, to facilitate replications and to ensure that null results are also published, which, needless to say, will advance the field. Furthermore, the need to enhance clarity and define the key concepts of embodiment in language research has been highlighted (e.g., Friedrich et al., 2025), with comprehensive definitions related to the field proposed in several overviews (e.g., Körner et al., 2023; Stockner et al., 2025).

In the current study, we examine mental simulation of speed-induced language by using the Finno-Ugric language Estonian as a testbed. This will enable the assessment of mental simulation in a structurally different language than English, the language typically examined in previous studies. Estonian features rich morphology, extensive use of case endings and postpositions rather than prepositions, and relatively free word order (Erelt, 2003). At the same time, it shares certain typological characteristics with English in terms of the neutral word order SVO (Lindström, 2017, p. 547) and general manner salience in expressing motion (Taremaa et al., 2024).

We use the term ‘mental simulation’ to refer to sensorimotor simulations evoked during language processing, that is, neural and bodily responses to language that arise when actually perceiving or conducting the same activities (see also Körner et al. 2023). We do not directly access the brain regions or brain activity. Instead, we assess eye-movements during a listening and comprehension task under the assumption that eye-movements can serve as a behavioural proxy for the sensorimotor processes engaged during language comprehension, established as such in studies of Speed and Vigliocco (2014) and Lindsay et al. (2013). Furthermore, we focus on language processing only and do not look into mental simulation as envisioning or conscious imagery (e.g., Cole et al. 2021).

### 1.1 The Complex Nature of Mental Simulation

A range of behavioural studies have provided evidence for the existence of mental simulation and eye-movements can serve as a behavioural proxy for the sensorimotor processes engaged during language comprehension. For instance, Castaño and Carroll (2020) found both literal and metaphorical expressions trigger eye-movements upwards or downwards dependent on the direction expressed in a sentence (e.g., *The curtain is rising* and *The amount is rising*; for similar early results on direction effects, see Spivey and Geng (2001)). In this experiment, participants listened to sentences, while looking at a blank screen divided into four equal squares. It was found that the direction expressed by the verb in the sentences (both in literal and metaphorical sentences) influenced the gaze position, directing it to the same direction during the post-verb period.

Spatial simulation can also extend to the domain of time and evoke mental simulation, as shown by Stocker et al. (2016) in German. In their study, people listened to two sentences while their eye-movements to a blank screen were recorded. The first sentence described current time (e.g., *Now I’m watching TV*), and the second sentence described either the past (e.g., *Before that I was listening to musi*c), present (e.g., *At the same time I’m listening to music*), or future (e.g., *After that I will be listening to musi*c). The study revealed a significant effect of time on eye-movements along the vertical axis. People made more upward saccades in response to future time descriptions compared to past descriptions.

While there is a wealth of evidence for mental simulation, findings do not always replicate (Morey et al., 2022; Zwaan & Pecher, 2012), and the reasons for mixed results or non-replication may be linked to various factors. Importantly, mental simulation is context-dependent (Ibáñez et al., 2023), may not occur automatically in all situations (Von Sobbe et al., 2021) and for all perceptual modalities (Speed & Majid, 2018), may flexibly and gradually unfold over time (Hoeben Mannaert et al., 2019; Liu et al., 2024; Pan et al., 2024; Speed & Vigliocco, 2014), may be influenced by processing depth (Connell, 2019; Vukovic et al., 2017), and may be susceptible to individual variation (Ibáñez et al., 2023; Winter et al., 2022).

Finally, existing research on mental simulation, and in cognitive science more generally (Blasi et al., 2022), is also limited by its Indo-European language-bias. For instance, a meta-analysis of studies that closely followed the original study of action-sentence compatibility effect (ACE; Glenberg & Kaschak, 2002) included only English, German, Spanish, Italian, and French, with English being the most researched in the experiments covered by the meta-analysis (Winter et al., 2022). Similarly, a large multi-lab replication project of the ACE by Morey et al. (2022) was based on English-speaking participants only. In addition, given the difficulties in replication in mental simulation research (which, in turn, can be related to methodological issues rather than evidence against mental simulation) and general publication bias (Rosenthal, 1979), there is an increasing need for replication and pre-registered studies (see also Morey et al., 2022).

### 1.2 Mental Simulation of Speed-induced Language

One aspect of mental simulation that remains underexplored, is the extent to which fine-grained motion information, such as speed, is included in mental simulations. Speed is an integral part of everyday human experience, routinely expressed in language. Therefore, processing speed-induced language should evoke traces of embodiment. At the same time, speed is a complex phenomenon that combines both space and time, making it challenging to measure mental simulation when processing speed language. Furthermore, when expressed in language, speed does not solely characterise physical motion, but extends to more abstract domains, possibly through the conceptual metaphor action speed is motion speed (Lahlou, 2023; Lakoff et al., 1991).

One way to measure the simulation of speed language is through language processing speed, such as reaction times, i.e., reducing speed simulation to temporal processes. If automatic simulation is evoked in response to language describing fast action/motion (e.g., the verb *run*) versus language describing slow action/motion (e.g., the word *walk*), speakers should respond to fast language more quickly than slow language, which, in turn, has been interpreted as an indication of comprehension speed. This expectation has been tested in several studies by measuring the speed at which speakers process lexemes or sentences conveying either fast or slow speed.

For example, Pan et al. (2024) found that Mandarin Chinese fast verbs are processed more quickly than slow verbs, whether participants need to distinguish them from pseudo-verbs or to evaluate their similarity to other verbs. Ben-Haim et al. (2015) showed that in Hebrew, naming vehicles of fast motion (e.g., airplane) and assigning speed ratings to them took less time than naming or evaluating vehicles of slow motion (e.g., wheelchair). They found the same effect for reading in that reading latencies for vehicle words were shorter for fast than for slow vehicles. If speed was implied by the context rather than by the characteristics of the vehicle (e.g., a car going up or down the hill), naming latencies were again shorter for contexts supporting fast motion (e.g., moving downhill) and longer for slow motion (e.g., going uphill).

Von Sobbe et al. (2021) found a similar result in German sentence processing where verb speed was varied in the sentences (e.g., *sprinten* as a fast verb and *wandern* as a slow verb) in that people were faster in evaluating the motion content of the sentences with fast than with slow verbs. Importantly, they also demonstrated that task-related factors may hinder this effect, as when the task was to evaluate whether the sentence was semantically acceptable, the slow sentences were evaluated faster than the fast sentences.

Stites et al. (2013) demonstrated that in the sentential context of direct and indirect quotation, fast adverbs (e.g., *John walked into the room and said* energetically…) were read faster than slow adverbs (e.g., *John walked into the room and said* nonchalantly…) in English, as evidenced by eye movement data. This effect was observed regardless of whether the adverbs were used in combination with fast or slow verbs (e.g., *bolt* and *walk* respectively). In contrast to Pan et al. (2024), who found differences between fast and slow verbs in Chinese, no significant differences were found in the reading times of fast and slow verbs. Similarly, Irie et al. (2021) found that in Japanese, sentences of fast vs. slow movements of the hand were evaluated for their meaningfulness more quickly when they contained a word meaning ‘fast’ or ‘quick’ than when they included a word meaning ‘slow’. Interestingly, the same effect was not found for sentences describing leg movement, which supports earlier evidence of speed simulation for hand actions but not leg actions (Speed et al., 2017).

Another way to measure the simulation of speed is through its spatial characteristics, reducing speed simulation to the processing of spatial information. If simulation takes place, the locations of entities moving at a particular speed should be processed. This can be assessed through attention allocation experiments, particularly when combined with eye-tracking methodology. Using this approach, Richardson and Matlock (2007) found that fictive motion expressions implying slow motion (motion along a difficult terrain, as expressed, for example, by *The valley is covered with ruts*, and followed by the fictive motion expression *The road runs through the valley*) compared to fast motion (motion along an easy terrain, as in *The valley is covered with dust*, followed by *The road runs through the valley*) led to increased attention to the path of motion in the visual field. Similarly, Lindsay et al. (2013) demonstrated that slow-motion sentences elicited more attention to the path of motion, whereas fast-motion sentences directed attention more toward the endpoint of the motion. On the contrary, Speed and Vigliocco (2014) found that slow-motion elicited more attention toward the destination of an Agent in motion compared to fast motion sentences.

Finally, when examining speed, one can attempt to measure it as a combined category of space and time, which inevitably means that both spatial and temporal aspects must be considered. This means that online processing over temporal and spatial domains has to be measured, as can be done, for example, through time-course analyses of eye-tracking experiments (Lindsay et al., 2013; Speed & Vigliocco, 2014), while also taking into account that mental simulation of language is not invariant, but instead flexibly changes in response to varying circumstances (Hoeben Mannaert et al., 2019).

Taken together, research on the mental simulation of speed-induced language suggests mixed findings. The realisation of such simulations can sometimes also be inferred from contrastive outcomes (compare Lindsay et al., 2013; Speed & Vigliocco, 2014). This raises questions about the granularity of speed in mental simulation and more generally about when a sensorimotor simulation is necessary for comprehension and when it is not, and about which measurement tools can be used to establish such simulations.

### 1.3 The Conceptual Replication of the Simulation of Speed

The current study builds on previous investigations of speed simulation in two ways: 1) We extend this research beyond Indo-European languages to Estonian, and 2) We conduct two eye-movement studies in an attempt to disentangle contrasting findings by Speed and Vigliocco (2014) and Lindsay et al. (2013) that were conducted in English. Through this, we evaluate the replicability of mental simulation research and the extent to which mental simulation is observed across languages. Speed and Vigliocco (2014) and Lindsay et al. (2013) investigated whether mental simulation of speed occurs, with a goal to reveal how fine-grained mental simulation is. Interestingly, although the studies were very similar in design, they gave somewhat contradictory results. In both studies, participants were presented with pictures accompanied by auditory sentences describing the scenes, while their eye-movements were recorded. The sentences expressed Goal-directed motion and varied in verb semantics, such that half of the verbs conveyed fast and the other half slow motion (e.g., *dash* and *amble* respectively). The corresponding pictures depicted an Agent, Path, and Goal.

Speed and Vigliocco (2014) hypothesised that the duration of simulation should be affected by the speed implied by the sentence, such that fast actions would result in shorter looking times to objects compared to slow actions. Lindsay et al. (2013) hypothesised that because fast motion would result in reaching a Goal more quickly, fast verbs would elicit more and earlier attention to the Goal, while slow verbs would lead to greater attention to the Path. Both studies found evidence supporting their respective hypotheses.

Speed and Vigliocco (2014) found that the effect of verb speed depended on the speech rate of the sentence. Verb semantics influenced looking times when the speech rate was slow but not when it was fast. In Experiment 1, when there was a distractor present in the scene, slow verbs increased the total dwell time on the Agent but not on the Goal, whereas in Experiment 2, when no distractor was present, slow verbs increased the total dwell time on the Goal, and not on the Agent. In Experiment 3, where speech rate was not manipulated but half of the visual scenes included distractors, longer looking times on the Goal were observed in response to slow verbs, but only in scenes without a distractor. In contrast, Lindsay et al. (2013) found that sentences with slow verbs led to longer total dwell time on the Path, while sentences with fast verbs led to longer dwell time on the Goal. No significant effects were observed for looks to the Agent. The contradictory finding lies in the Goal-related results: while Speed and Vigliocco (2014) reported longer looking times on the Goal in response to slow verbs, Lindsay et al. (2013) found the opposite in that fast verbs resulted in longer looking time on the Goal. When looking at the time course data however, results of the two studies converged. That is, the average proportion of fixations towards the Goal over time was delayed for slow-verb sentences as compared to fast-verb sentences.

Even though the original studies by Speed and Vigliocco (2014; Experiment 3) and Lindsay et al. (2013) were very similar in design and administration, they also had notable differences. In Speed and Vigliocco (2014), the sentences were in the past tense and included an Agent, verb, and Goal (e.g., *The lion dashed to the balloon*). In contrast, sentences in Lindsay et al. (2013) were in the future tense and consisted of an Agent, verb, Path, and Goal (e.g., *The bear will dash along the trail to the tent*). Speed and Vigliocco (2014) used pictures both with and without distractors (referred to as double and single scenes, respectively), which depicted horizontal or diagonal paths. Lindsay et al. (2013), on the other hand, used only pictures without distractors, with an equal number of horizontal and vertical paths and their paths were more visually salient (i.e., wider). In addition, eye-movements were recorded from sentence onset in Speed and Vigliocco (2014) and from verb onset in Lindsay et al. (2013). For the total dwell time measure, Speed and Vigliocco (2014) included all fixations from sentence onset until the end of the trial, whereas Lindsay et al. (2013) included only the fixations occurring between verb onset and sentence offset. Furthermore, the data was analysed using linear mixed-effects models in Speed and Vigliocco (2014), while Lindsay et al. (2013) applied Generalized Estimating Equations (GEE). Finally, comprehension questions were presented after each trial in Speed and Vigliocco (2014), whereas in Lindsay et al. (2013), participants were only instructed to listen carefully, with no additional tasks. Because mental simulation is flexible and context-dependent, Speed and Vigliocco (2014, p. 380) suggest that the diverging results may be attributed to differences in the composition of the linguistic and visual stimuli.

### 1.4 The Current Study

We use the same design, visual stimuli, and procedure as Speed and Vigliocco (2014), with the language stimuli replaced by their Estonian equivalents in Experiment 1, and modified as inspired by the study of Lindsay et al. (2013) in Experiment 2. This modification entailed lengthening the linguistic stimuli by including a Path phrase, whereas all other components of the experiment remained as in Experiment 1 (i.e., as in Speed and Vigliocco (2014)). Both experiments were pre-registered on the OSF (Experiment 1: https://osf.io/wqf7e; Experiment 2: https://osf.io/4dp9k). Estonian as a Finno-Ugric language makes it possible to iterate the experiment previously conducted in Indo-European languages quite precisely, while also extending the study to a language that differs from Indo-European languages in several distinct ways. Differently from English, Estonian has relatively free word order (with SVO order being the most neutral one), the absence of articles before nouns, a hybrid system of case endings, prepositions, postpositions, and particles (with cases and postpositions being used often where English, for example, would use prepositions), and the lack of a grammatical future tense. Similarly to English, speed as a manner dimension can be conveyed by motion verbs, and characteristically to a satellite-framed manner-salient language (Slobin 2006), such verbs are productively used in Estonian (Taremaa et al. 2024).

In Experiment 1, we hypothesise that the results for Estonian will echo those for English in the original study of Speed and Vigliocco (2014). Specifically, we expect an interaction between speed of the verb and scene type on looks to the Goal. Our pre-registered hypothesis was that the total dwell time on the Goal should be longer for sentences with slow verbs than for sentences with fast verbs in single scenes, but not in double scenes.^1^ An alternative outcome would be the reverse effect, with longer looking times on the Goal for fast verbs compared to slow verbs, as reported by Lindsay et al. (2013), and this is what we expected for Experiment 2 when the linguistic stimuli mentioned a Path, as in Lindsay et al. (2013). Our pre-registered hypothesis for Experiment 2 was that the Goal will receive longer and the Path shorter looking times in response to fast motion descriptions compared to slow motion descriptions. Consistent with both studies, we also pre-registered the hypothesis in Experiment 1 that listeners will direct their gaze to the Goal more quickly when hearing sentences with fast verbs than with slow verbs.

## 2. Experiment 1

In Experiment 1 we used the same experimental procedure and design as Experiment 3 in Speed and Vigliocco (2014).

### 2.1 Method

#### 2.1.1 Design and Stimuli

As in Speed and Vigliocco (2014), the experiment had a 2 × 2 within subject design with the factors Verb Speed (fast vs. slow) and Scene type (single vs. double). Sixteen sentence pairs were created. Sentences were in the past simple tense and conveyed motion of an animate figure (Agent) to a destination (Goal). The Goal was always referred to with a postpositional phrase with the postposition *juurde* ‘to’. When reporting Goal (phrase) onset in the analyses, this refers to the onset of the first word of the phrase, i.e. noun. Each sentence had two versions: one with a motion verb of fast motion, as in (1), and one with a motion verb of slow motion, as in (2). Verb semantics was confirmed in a separate rating study with 37 participants, where the motion verbs of the experiment were rated for how fast or slow the motion expressed by them is. Fast verbs received a mean rating of 7.5 (*SD* = 0.85) and slow verbs 2.4 (*SD* = 0.66) on a continuous scale from 0 to 10.

  (1)
*[Laps]_Agent_*
    child.nom
*[vudis]_fast_verb_*
    scurry.pst.3sg
*[puu*
    tree.gen
*juurde]_Goal_*
    to
    ‘The child scurried to the tree.’

  (2)
*[Laps]_Agent_*
    child.nom
*[tatsas]_slow_verb_*
    toddle.pst.3sg
*[puu*
    tree.gen
*juurde]_Goal_*
    to
    ‘The child toddled to the tree.’

Sentences were recorded by a native female speaker of Estonian. To maintain consistency across sentence pairs of slow and fast motion, the verb was cross-spliced, with the source sentence the same in the fast and slow versions. In addition, 32 filler sentences were created and recorded. Filler sentences contained an Agent acting upon another figure (e.g., *Koer urises palli peale* ‘The dog growled at the ball’) or, in combination with a general motion verb, an Agent and a Goal phrase (e.g., *Karu läks mäe juurde* ‘The bear went to the mountain’).

**Figure 1:**
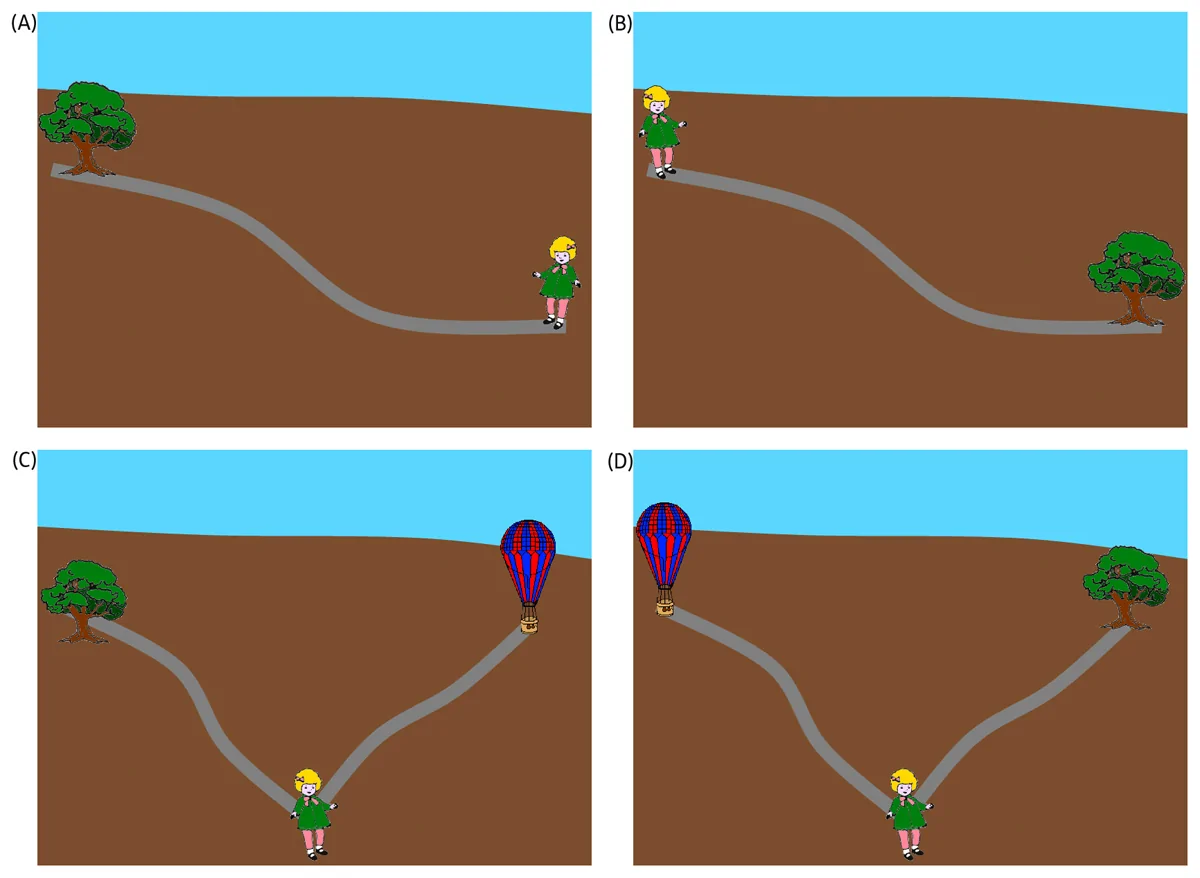
Examples of the Four Versions of the Picture Stimuli. *Note*. The panels correspond to the sentence pair ‘The child scurried/toddled to the tree’, standing for the four image versions: **A)** Single scene, left Goal, **B)** Single scene, right Goal, **C)** Double scene, left Goal, **D)** Double scene, right Goal.

Each sentence pair had a matching image containing a mover (Agent) and a destination entity (Goal), connected by a narrow path (Path). Half of the pictures also included distractor goals. Each picture had four versions, with counterbalanced position of the Goal to the left and to the right across single scenes (without distractor) and double scenes (with distractor), as shown in Figure 1. Images were taken from Speed and Vigliocco (2014).

#### 2.1.2 Participants

A total of 40 native speakers of Estonian were recruited, as set out in the pre-registration, in an effort to closely follow the decisions of the original study of Speed and Vigliocco (2014). For that, 51 adults voluntarily participated in the experiment, of whom 40 are included in the data analysis (32 female, 5 male, 3 non-binary; mean age 26.7 years). Eleven participants were excluded either because their accuracy in responding to comprehension questions was lower than 80% (6 participants), because their data did not include looks to the interest areas (IAs; missing data on IAs more than 17%; 4 participants), or due to a technical error by which eye-movements were not saved (1 participant). Participant exclusion was established in the pre-registration. The exclusion threshold for missing data in the eye-tracking measures was decided during data inspection, following common practices. All participants had normal or corrected to normal vision (in that case, they wore contact lenses). Participants were reimbursed with gift cards of 5 euros (or, upon choice, chocolate).

#### 2.1.3 Procedure

The experiment was conducted in the Phonetics lab at the University of Tartu, using SR Research EyeLink 1000+ eyetracker, desktop mount, a chin-rest, and a head-rest. The participants’ right eye was tracked with a sampling rate of 1000 Hz. Before the experiment, participants were briefed about the experiment and their task; participants also signed the consent form and filled in a short background survey. A 9-point calibration was performed before and halfway through the experiment. In the experiment, participants were presented with pictures and the corresponding audio sentences describing the pictures.

First, a black circle on white background was displayed on the screen for drift correction. Then, a picture appeared on the screen. After a 1000 ms delay, the audio was played while the picture remained on the screen. Mean length of the audio was 2238 ms (*SD* = 170 ms, range 1787–2481 ms). After the end of the audio, the picture remained on the screen for additional 1500 ms, after which a comprehension question was displayed, written on the white screen.

Participants were instructed to listen to the sentences describing the pictures and respond to comprehension questions by pressing the left button of the mouse if the answer was ‘no’, and the right button if the answer was ‘yes’. There were six practice trials, followed by experimental trials containing 32 test items and 32 filler items. Items were presented in a randomised order and in two blocks with an opportunity to take a break between the two blocks. Gaze-movement data and responses to the comprehension questions were recorded. The whole procedure and design followed what was outlined in the pre-registration to closely match the original study by Speed and Vigliocco (2014).

### 2.2 Results

#### 2.2.1 Data Pre-processing

Following Speed and Vigliocco (2014), and as pre-registered, fixations shorter than 150 ms were removed. Prior to exclusion, short fixations within the distance of 0.5 visual angle were merged, following the default four stage data cleaning settings of DataViewer; 21% of the fixations were removed.

After merging and cleaning the data, 10268 fixations remained, counted from audio onset to the end of trial. Of these, 4298 (42%) fell on the Agent, 516 (5%) on the Path, and 3229 (31%) on the Goal IAs. Outside the IAs, 1013 (10%) fixations landed on the Distractor and 1212 (12%) elsewhere in the scene. In the analyses, fixations outside the Agent, Path, and Goal IAs were not considered. In addition, a total of 840 units of track loss (whether because of blinks or other reasons; all labelled as blinks by the software) occurred within trials, separating consecutive fixations (regardless of their spatial proximity). Blinks were excluded from the dataset during data export from DataViewer; hence, the final dataset contains no missing data. Participants did not fixate on all IAs in every trial. Of the 1280 trials in total (32 trials for each 40 participants), the Agent was fixated in 1257 trials (98%), Goal in 1195 trials (93%), and Path in 335 trials (26%). Per participant, the number of trials with fixations on the Agent ranged from 29 to 32 (*M* = 31.4, *SD* = 0.8), on the Path from 3 to 23 (*M* = 8.4, *SD* = 4.4), and on the Goal from 23 to 32 (*M* = 29.9, *SD* = 2.6). The proportion of trials across conditions where IAs were fixated is presented in Table 1. In our statistical modelling, the total dwell time is only represented for trials where the respective IA had fixations.

**Table 1:** Fixation Coverage of Each Interest Area (IA) across Conditions (Verb Speed × Scene) in Experiment 1.


SCENE	VERB SPEED	AGENT	PATH	GOAL

Single	Fast	315 (98.4%)	132 (41.2%)	307 (95.9%)

Single	Slow	316 (98.8%)	115 (35.9%)	306 (95.6%)

Double	Fast	312 (97.5%)	43 (13.4%)	286 (89.4%)

Double	Slow	314 (98.1%)	45 (14.1%)	296 (92.5%)


*Note*. The table shows the number and proportion of trials in which the IA was fixated, averaged across participants. Each condition included 320 trials (40 participants × 8 items per condition).

The total dwell time was calculated for each IA as a sum of fixation durations on the respective IA across the trial. In the pre-registration, only the IAs for Agent and Goal were included. However, to compare the results with Experiment 2 where Path was also included as an IA, it was deemed necessary to include Path in the current analyses as well. In the pre-registration, the time window was not specified. To ensure the results are comparable to those of the original studies, two time windows as interest periods were created. Consequently, a wide time window was defined similarly to Speed and Vigliocco (2014) as a period from sentence onset until trial offset (i.e., until the end of additional 1500 ms after sentence offset when the picture remained on the screen). A somewhat narrower time window was defined similarly to Lindsay et al. (2013) as a period from verb onset until the sentence offset.

For the time-course analysis, the data was extracted from Data Viewer as a sample report, providing gaze information for every millisecond of the trials. 2.5% of the samples show missing data (blinks or trackloss) and were excluded during subsequent preprocessing. The sample report data contains all fixations, including those falling outside IAs. The pre-processing method for the time-course data, the modelling method, and the model structure were not specified in the pre-registration, but were decided later based on the existing literature. The data was pre-processed using the package VWPre (Porretta et al., 2016), following the procedure outlined by Porretta et al. (2018) and Porretta (2019). 50 ms bins were used, starting from the sentence onset and finishing at the offset of the trial. Proportions of fixations towards the IAs were calculated and transformed to empirical logits to enable modelling of them as dependent variables. Since dependent variables should not be bounded, as proportions are, it is common practice to use log-scaled proportions, which can vary from negative to positive infinity (see also Barr, 2008; Porretta et al., 2018). In addition, to capture the differences in fixations towards the IAs, the variable of Goal–Agent fixation difference was created by subtracting the empirical logits of the Agent IA from the Goal IA. Rather than addressing the fixations on one IA, this approach, common in similar eye-tracking studies, allows for considering fixations on a given IA in relation to fixations on other IAs. We use this variable for time course analyses. The supplementary material on the OSF provides analyses also for the Goal IA only, with no differences in the results.

#### 2.2.2 Total Dwell Time

For the total dwell time, linear mixed effects models were fitted with the same structure as in Speed and Vigliocco (2014), separately for Goal, Agent, and Path. Because the total dwell time was positively skewed, it was log-transformed. The log-transformed total dwell time was modelled as a function of the interaction between Scene and Verb Speed, with random intercepts for item and participant. A simple random-effects structure was chosen in line with the analysis by Speed and Vigliocco (2014) and as pre-registered (post hoc attempts with more complex random-effects structures failed to converge). All models included Verb Duration (ms) and Verb Frequency (log10) as covariates to control for potential confounds. R package lme4 (Bates et al., 2015) was used for modelling with the lmer() function. Significance of fixed effects was assessed using likelihood ratio tests comparing the full model to reduced models omitting each effect of interest, as implemented in the mixed() function from the afex package (Singmann et al., 2024). Post-hoc contrasts were decided during data analyses (i.e., they were not pre-registered). They were assessed using the estimated marginal means (EMMs), following the suggestions of Garofalo et al. (2022) and using the emmeans package (Lenth 2025).

Because the two time windows (that used in Speed and Vigliocco (2014) and that used in Lindsay et al. (2013)) yielded similar results, only the analyses of the wide time window are presented here (from sentence onset until trial offset). The modelling results for the narrow time window can be found in Appendix A and in the supplementary materials on the OSF.

**Figure 2:**
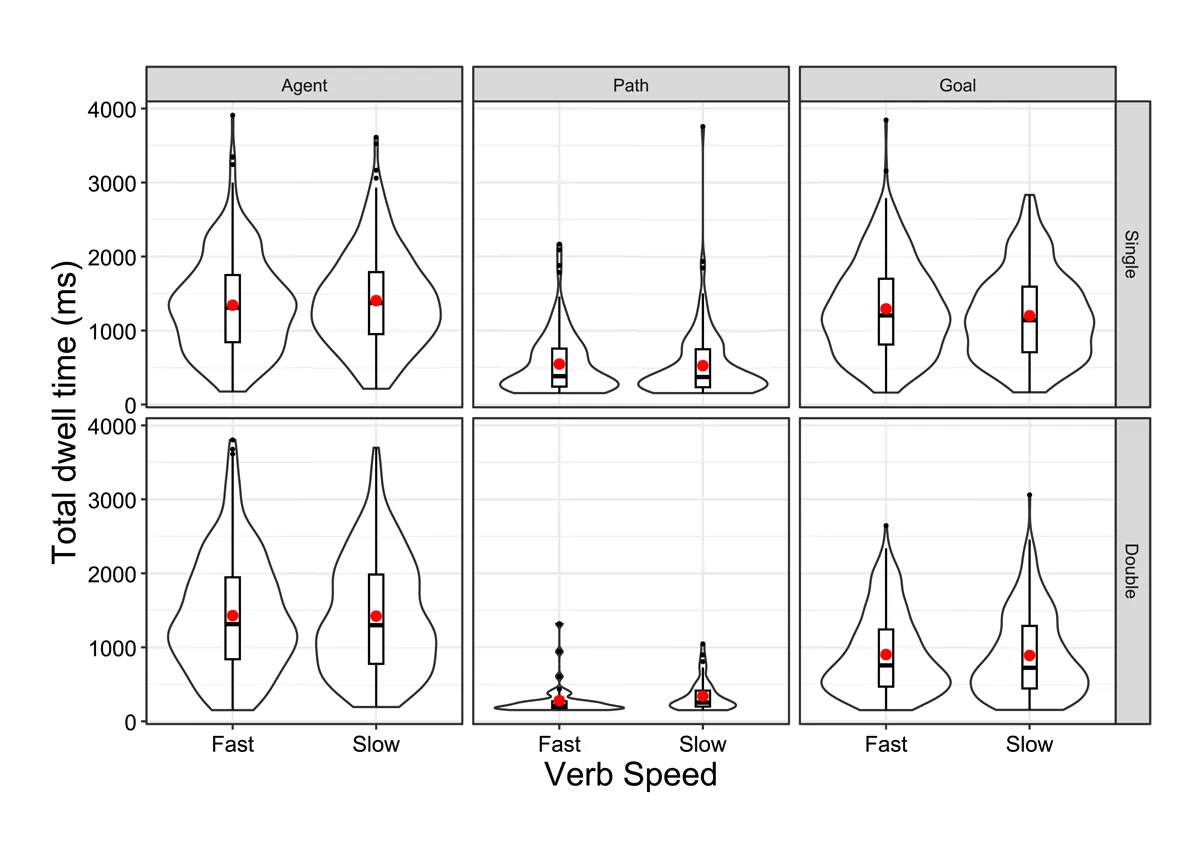
Average Total Dwell Times in Experiment 1. *Note*. Violin plots show the distribution of the total dwell times on the IAs in single scenes (upper panel) and double scenes (lower panel) calculated across the whole trial starting from sentence onset. Red dots stand for the mean and horizontal lines for the median.

Mean total dwell time is depicted in Figure 2. Contrary to our hypotheses, neither Verb Speed nor the interaction between Verb Speed and Scene was significant in any of the models (see Table 2; for model estimates, see Appendix A). For dwell time on the Agent in single scenes, the estimated difference between fast and slow conditions was –0.06 (95% CI [–0.21, 0.09]) and in double scenes –0.008 (95% CI [–0.16, 0.15]). In the Path and Goal model, there was a significant effect of Scene. This merely indicates that in double scenes, there were more items people could attend to (hence, less time to fixate on), and the length of the Path figures was shorter than in single scenes. This is also reflected in IA coverage in terms of trials where IAs were fixated, with Path featuring a substantially low proportion of looks (41% and 36% in single scenes and 13 and 14% in double scenes; see Table 1). The estimated difference for looks to the Path between fast and slow was 0.05 (95% CI [–0.12, 0.21]) in single scenes and –0.20 (95% CI [–0.46, 0.07]) in double scenes. For looks to the Goal, the estimates were 0.07 (95% CI [–0.07, 0.21]) in single scenes and 0.02 (95% CI [–0.12, 0.16]) in double scenes. In sum, results on the total dwell time do not provide support for the hypothesis that verb speed affects eye movement patterns. However, because Path is infrequently fixated, the results for this IA should be interpreted with caution, and because the confidence intervals are wide, the overall results should be interpreted as inconclusive.

**Table 2:** Significance Tests for LMER Models in Experiment 1 (Wide Time Window).


EFFECT	AGENT MODEL			PATH MODEL			GOAL MODEL		
		
	*df*	χ²	*p*	*df*	χ²	*p*	*df*	χ²	*p*

Verb Speed	1	0.69	.408	1	0.32	.569	1	1.12	.290

Scene	1	0.04	.835	1	27.12	<.001	1	80.61	<.001

Verb Frequency	1	6.46	.011	1	4.55	.033	1	0.51	.475

Verb Duration	1	1.83	.176	1	2.44	.119	1	1.52	.218

Verb Speed × Scene	1	0.70	.404	1	2.45	.118	1	0.63	.427


To assess whether the observed null results indicate inconclusive evidence or evidence favouring models excluding the effect of interest, we calculated Bayes Factors (BF) using the BayesFactors package (Morey & Rouder, 2026), following recent suggestions in the literature (Dienes 2019). This analysis was not part of the pre-registration, but appeared necessary to evaluate the modelling results. We report BF_10_ values, which indicate the relative evidence for the model including the variable of interest compared with the model without it; for the interaction, the comparison was made between the model with the interaction term and the model with the corresponding main effects. The full analysis across priors with different sensitivities (0.3, 0.4 and 0.5) is available on the OSF. Importantly, if the BF_10_ value is smaller than ⅓, there is evidence in favour of the model excluding the effect of interest. If the value is larger than 3, this indicates evidence in favour of the model including the effect. If the value falls between the two, the evidence is inconclusive. Across all sensitivity scenarios, BF_10_ values for Agent and Goal models provide evidence favouring the models excluding the effect of Verb Speed over the models including it. For Agent, BF_10_ values for Verb Speed as the main effect ranged from 0.18 to 0.26 and for the Verb Speed × Scene interaction from 0.12 to 0.21; for Goal, BF_10_ values ranged from 0.16 to 0.26 for Verb Speed and 0.14 to 0.25 for the interaction. For Path, BF_10_ values for the main effect of Verb Speed ranged from 0.19 to 0.29, providing evidence in favour of models that excluded the effect; BF_10_ values for the interaction were inconclusive, ranging from 0.66 to 0.86.

#### 2.2.3 Time-course Analysis

**Figure 3:**
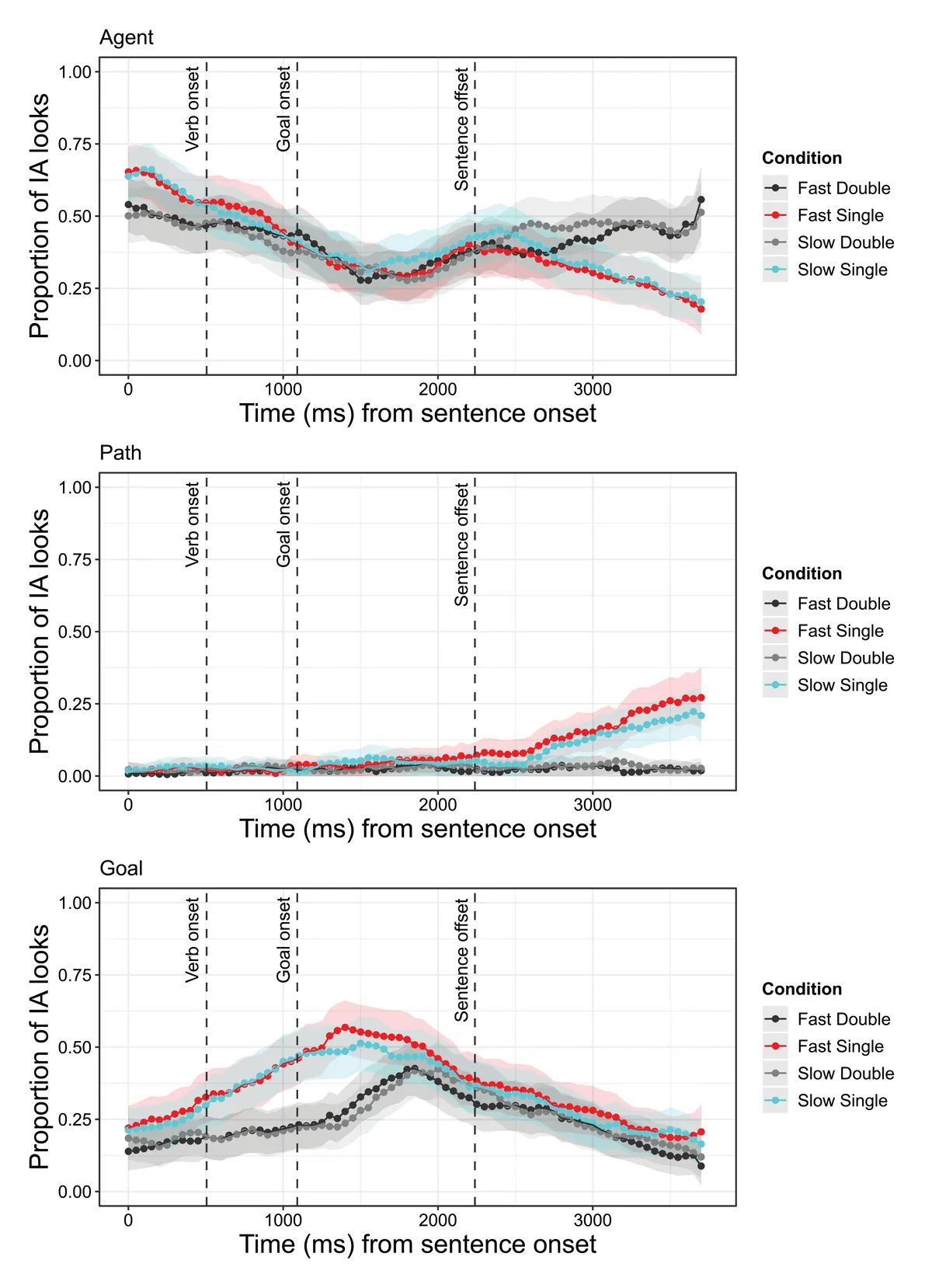
Grand Averages of the Proportions of Looks to the IAs across the Trial Time Starting from Sentence Onset in Experiment 1. *Note*. The mean verb onset was 505 ms (*SD* = 64, range 395–655 ms) and Goal phrase onset was 1091 ms (*SD* = 82 ms, range 875–1294 ms). Shaded areas indicate 95% confidence intervals.

Visualising the looking patterns, the figure of grand averages (Figure 3) indicates no great differences between fast and slow conditions within single and double scenes. Only modestly, in single scenes around 500 ms after verb offset (which corresponds to the Goal phrase onset), the Agent receives somewhat more looks in response to slow verbs than to fast verbs. Conversely, after around 200 ms of verb offset, the Goal receives more looks in response to fast than to slow verbs.

**Figure 4:**
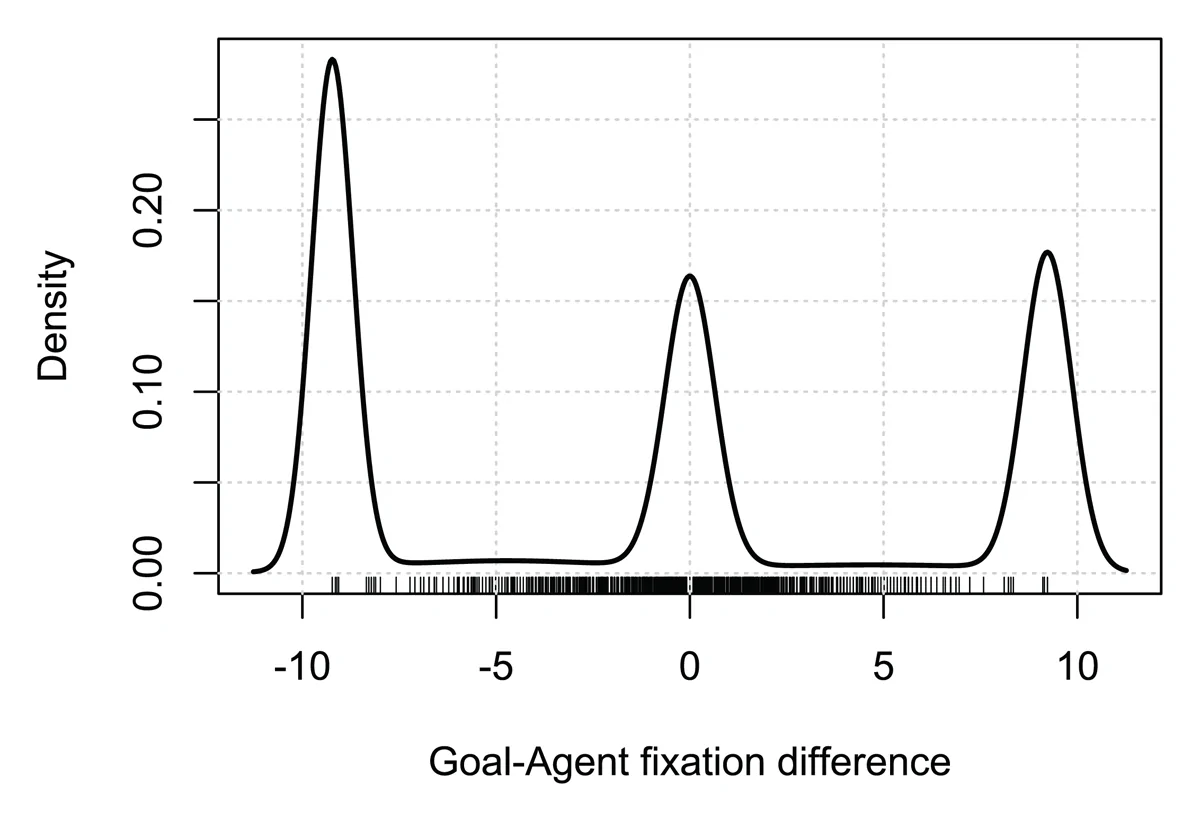
Density Plot of the Goal–Agent Fixation Difference in Experiment 1.

For a more fine-grained analysis, and to measure the change of eye-movement patterns across the trial, generalised additive mixed models (GAMMs) were fitted using the mgcv package (see also Wood, 2011, 2017, 2025). For visualisation, the itsadug package was used (van Rij et al., 2022). In the pre-registration, we did not specify the statistical method for time-series data. We chose GAMMs because they allow modelling time-course data which is typically non-linear, while also taking into account the correlations between consecutive time bins (see also Ito & Knoeferle, 2022). As a response variable, the difference between the proportions of fixations on the Goal and Agent IA was used (Goal–Agent fixation difference), subtracting the Agent empirical logits from the Goal empirical logits. Thus, we model Goal preference, following the procedure of similar visual world paradigm studies (e.g., Blything et al., 2021). Positive values of the Goal–Agent fixation difference stand for preference for the Goal, negative for Agent, and 0 no preference in that either both Goal and Agent received an equal proportion of looks, or neither did (see also Figure 4).

Condition as an interaction term between Verb Speed and Scene was used as the parametric response variable, with four levels: Fast Single (reference level), Slow Single, Fast Double, Slow Double. By-participant and by-item random smooths of time were added, as well as smooths for Verb Duration and Verb Frequency. In addition, the model incorporated weights equal to the inverse of the summed variances of the Goal IA and Agent IA empirical logits. Because fixations can extend across consecutive time bins, these bins may be highly correlated. To account for this autocorrelation, an AR1 correlation structure was included in the final GAMM model, with a rho value of .94. The time window for analysis extended from sentence onset to the mean trial offset.

The model summary (see Table 3) indicates that without considering time-variability, the parametric coefficients show that fast verbs in single scenes are significantly different from fast verbs in double scenes, which is not particularly informative for our purposes. It merely reflects differences in looks at sentence onset, when time equals zero. The condition varying by time, represented by the smooth terms (looking behaviour across time) is more informative, suggesting significance of Condition. Because the four-level condition codes Verb Speed and Scene interaction, these smooths also capture main effects of Verb Speed and relevant contrasts (e.g., Fast Single vs. Slow Single). As the *p* values of the smooth terms indicate, the looks over time significantly differed from zero and, as such, this indicates that there are differences in looks to Agent and Goal across the time-course of the trial.

**Table 3:** Summary of GAMM Results Predicting Goal–Agent fixation difference of Experiment 1.


A. PARAMETRIC COEFFICIENTS				

PREDICTOR	ESTIMATE	*SE*	*t*	*p*

(Intercept)	–0.229	0.297	–0.771	.441

Condition (Fast Double)	–1.351	0.287	–4.712	<.001***

Condition (Slow Double)	–1.345	0.394	–3.412	<.001***

Condition (Slow Single)	–0.336	0.394	–0.853	.394

**B. SMOOTH TERMS**				

**SMOOTH TERM**	***Edf***	***Ref. df***	***F***	***p***

s(Time): Condition (Fast Single)	6.669	7.587	8.419	<.001***

s(Time): Condition (Fast Double)	7.156	8.000	5.282	<.001***

s(Time): Condition (Slow Double)	7.063	7.939	4.961	<.001***

s(Time): Condition (Slow Single)	6.358	7.313	6.652	<.001***

s(Time, Subject) (random effect)	247.905	368.000	2.295	<.001***

s(Time, Item) (random effect)	160.550	284.000	1.352	<.001***

s(Verb Frequency)	1.021	1.028	3.163	.077

s(Verb Duration)	1.001	1.001	0.882	.348


*Note*. Model fit: Adjusted *R*² = .131; Deviance explained = 13.5%; *f*REML = 223,790; Scale estimate = 41.657; *n* = 99,502. *Edf* = estimated degrees of freedom. *Ref. df* = reference degrees of freedom. Significance codes: *** *p* < .001; ** *p* < .01; * *p* < .05.

**Figure 5:**
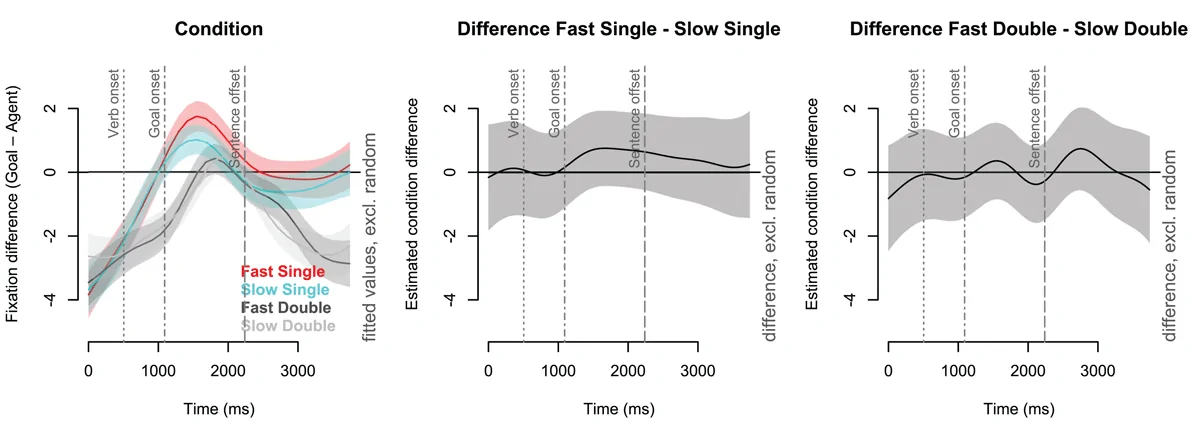
GAMM-estimated Smooths and Condition Differences in Experiment 1. *Note:* The smooths for the effect of Condition on Goal–Agent fixation difference (difference between the empirical logits of Goal and Agent) are on the left panel. GAMM-derived difference curves for the effect of Verb Speed on Goal–Agent fixation difference are shown for single scenes (middle panel) and double scenes (right panel). The x-axis is time in milliseconds, with 0 ms marking the onset of the audio.

Because the smooth terms indicate whether the time-course of fixations differs from zero, the effect of Condition itself (i.e., differences between conditions) is not directly interpretable from the table alone and requires a graphical companion. To further examine whether there are differences in the looking behaviour across time in response to fast and slow sentences, we use smooth and difference plots. Goal preference corresponds to larger Y-values and preference to other areas (mainly Agent) corresponds to smaller values (see Figure 5). Smooths for each time bin by Condition indicate that the Goal is fixated more in single scenes than in double scenes. Difference plots further show that there are no significant differences in single or double scenes between fast and slow sentences. Note that the results do not change if the zero timepoint is set to verb onset, nor when the dependent variable is Goal instead of the Goal–Agent fixation difference (see supplementary materials available on the OSF).

#### 2.2.4 Summary Experiment 1

In sum, eye-movement patterns did not differ significantly between fast and slow sentences, as evidenced by the pre-registered total dwell time and exploratory time-course analysis. The additional Bayes Factors (not pre-registered) provided evidence favouring the models excluding the main effect of Verb Speed for all IAs. The models excluding the Verb Speed × Scene interaction were favoured over the corresponding models including the interaction, apart from the Path IA, for which the evidence was inconclusive. Therefore, the results of Experiment 1 were not in line with the findings of previous studies. One possible reason for this outcome is that the stimuli were relatively short in duration. The mean duration of the sentences was 2238 ms, whereas in Speed and Vigliocco (2014), the mean duration was slightly longer at 2498 ms. It is possible that mental simulation may require more time to unfold, and the shorter duration may not have allowed sufficient time for this process to fully develop. To address this, we designed Experiment 2 to incorporate Path phrases into the linguistic stimuli, following the approach of Lindsay et al. (2013), in order to lengthen the sentences and provide more time for mental simulation to occur.

## 3. Experiment 2

### 3.1 Method

#### 3.1.1 Design and Stimuli

The design for Experiment 2 was the same as for Experiment 1. The only difference was in the structure of the linguistic stimuli that were lengthened by adding Path phrases, describing the motion of an animate mover along a Path to a Goal. This modification was made to allow more time for mental simulation to occur, and to reflect the sentence structure used in the second original study by Lindsay et al. (2013). However, to remain comparable to Experiment 1, we used linguistic stimuli in the past simple tense, though it diverges from Lindsay et al. (2013) who used future tense (note also that Estonian does not have the grammatical future tense). Each sentence had again two versions: containing a motion verb of fast motion, as in (3); and containing a motion verb of slow motion, as in (4).

  (3)
*[Laps]_Agent_*
    child.nom
*[vudis]_fast_verb_*
    scurry.pst.3sg
*[teed*
    path.part
*mööda]_Path_*
    along
*[puu*
    tree.gen
*juurde]_Goal_*
    to
    ‘The child scurried along the path to the tree.’

  (4)
*[Laps]_Agent_*
    child.nom
*[tatsas]_slow_verb_*
    toddle.pst.3sg
*[teed*
    path.part
*mööda]_Path_*
    along
*[puu*
    tree.gen
*juurde]_Goal_*
    to
    ‘The child toddled along the path to the tree.’

#### 3.1.2 Participants

We recruited 40 eligible participants, as outlined in the pre-registration and as in Experiment 1 for comparability. For that, 43 adults participated in the experiment, of whom 40 were included (23 female, 15 male, 2 non-binary; mean age 26.1), following the same criteria as in Experiment 1. Two participants were excluded for low comprehension question accuracy (lower than 80%). One participant was excluded because their eye-tracking data was not saved for technical reasons. No participants were excluded due to a large number of missing fixations on the IAs.

#### 3.1.3 Procedure

The procedure followed that of Experiment 1. Mean audio length was 3820 ms (*SD* = 216 ms, range 3416–4224 ms).

### 3.2 Results

#### 3.2.1 Data Pre-processing

Data pre-processing was done as in Experiment 1. For the total dwell time analysis, the fixation report was again extracted from Data Viewer after a four-stage cleaning process (19% of the fixations removed). From the 14426 fixations remaining after fixation merging and exclusion, 5960 (41%) fell on the Agent, 1150 (8%) on the Path, and 4361 (30%) on the Goal IAs. In addition, 1346 (9%) fixations fell on the Distractor and 1609 (11%) elsewhere in the scene. As in Experiment 1, only fixations on the Agent, Path, and Goal were considered, and blinks were excluded from the data (a total of 1471 blinks separating consecutive fixations occurred within trials). As for fixation distribution across the IAs, out of the 1280 trials (32 trials per 40 participants), the Agent received fixations in 1267 trials (99%), the Goal in 1244 trials (97%), and the Path in 548 trials (43%). Per participant, the number of trials with fixations on the Agent was 27–32 (*M* = 31.7, *SD* = 0.9), on the Path 5–21 (*M* = 13.7, *SD* = 4.2) and the Goal 26–32 (*M* = 31.1, *SD* = 1.2). The condition-based overview of IA coverage can be found in Table 4. For the time-course analysis, the sample report was used (3.9% of samples as blinks excluded) and preprocessed similarly to Experiment 1.

**Table 4:** Fixation Coverage of Each IA across Conditions (Verb Speed × Scene) in Experiment 2.


SCENE	VERB SPEED	AGENT	PATH	GOAL

Single	Fast	315 (98.4%)	203 (63.4%)	318 (99.4%)

Single	Slow	317 (99.1%)	210 (65.6%)	315 (98.4%)

Double	Fast	316 (98.8%)	60 (18.8%)	305 (95.3%)

Double	Slow	319 (99.7%)	75 (23.4%)	306 (95.6%)


*Note*. The table shows the number and proportion of trials in which the IA was fixated, averaged across participants. Each condition included 320 trials (40 participants × 8 items per condition).

#### 3.2.2 Total Dwell Times

**Figure 6:**
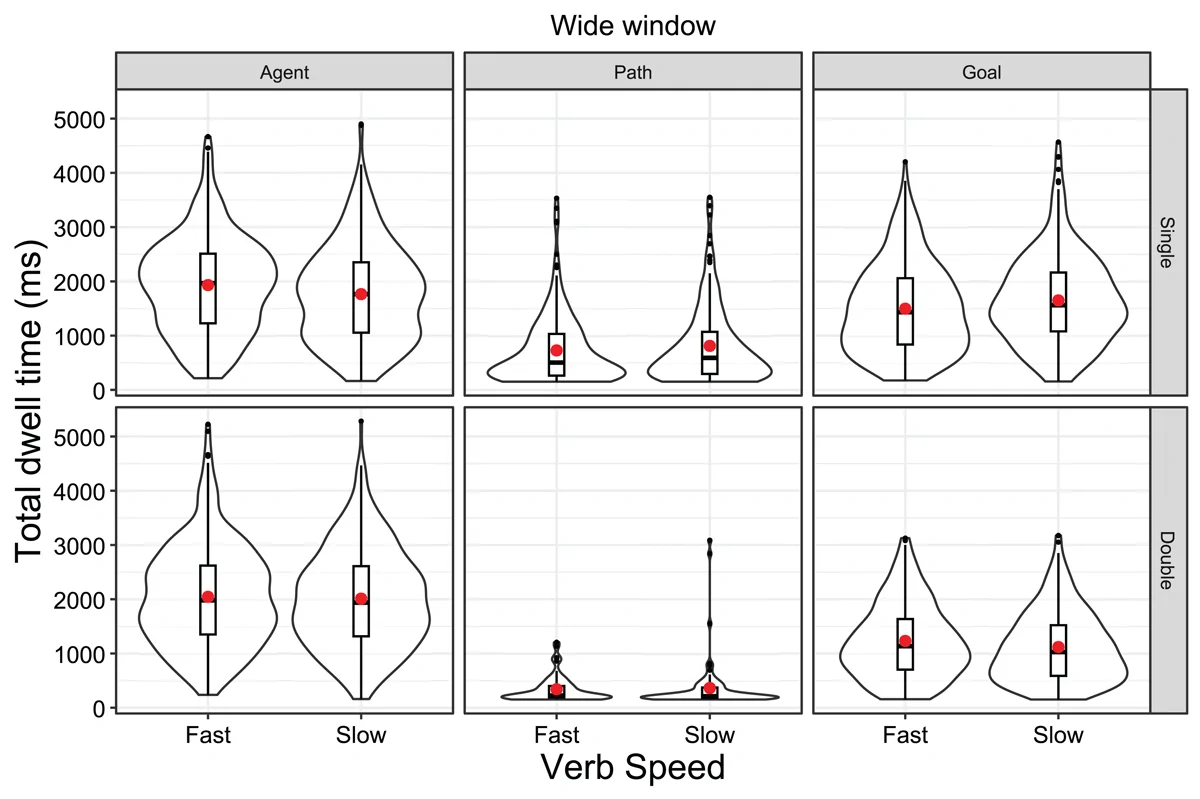
Average Total Dwell Times in Experiment 2. *Note*. Violin plots show the distribution of the total dwell times on the IAs in single scenes (upper panel) and double scenes (lower panel) calculated across the whole trial starting from sentence onset. Red dots stand for the mean and horizontal lines for the median.

The average mean total dwell times can be found in Figure 6. Following the pre-registration, the distributions of total dwell times were again evaluated in three separate linear mixed effect regression (LMER) models. The structure of the models was the same as in Experiment 1. The linear mixed effects models of the log-transformed total dwell time on Agent, Path, and Goal were fitted with Verb Speed, Scene and the Verb Speed × Scene interaction, as fixed effects (controlling for Verb Duration and Verb Frequency) and participant and item as random effects. Effect significances are presented in Table 5 (model estimates can be found in the Appendix A).

**Table 5:** Significance Tests for LMER Models in Experiment 2 (Wide Time Window).


	AGENT MODEL			PATH MODEL			GOAL MODEL		
		
EFFECT	*df*	χ²	*p*	*df*	χ²	*p*	*df*	χ²	*p*

Verb Speed	1	2.83	.092	1	0.89	.346	1	2.73	.099

Scene	1	3.18	.075	1	38.18	<.001	1	21.30	<.001

Verb Frequency	1	4.45	.035	1	0.39	.531	1	0.53	.465

Verb Duration	1	0.37	.540	1	0.47	.491	1	0.51	.474

Verb Speed × Scene	1	2.40	.121	1	0.51	.473	1	10.03	.002


**Figure 7:**
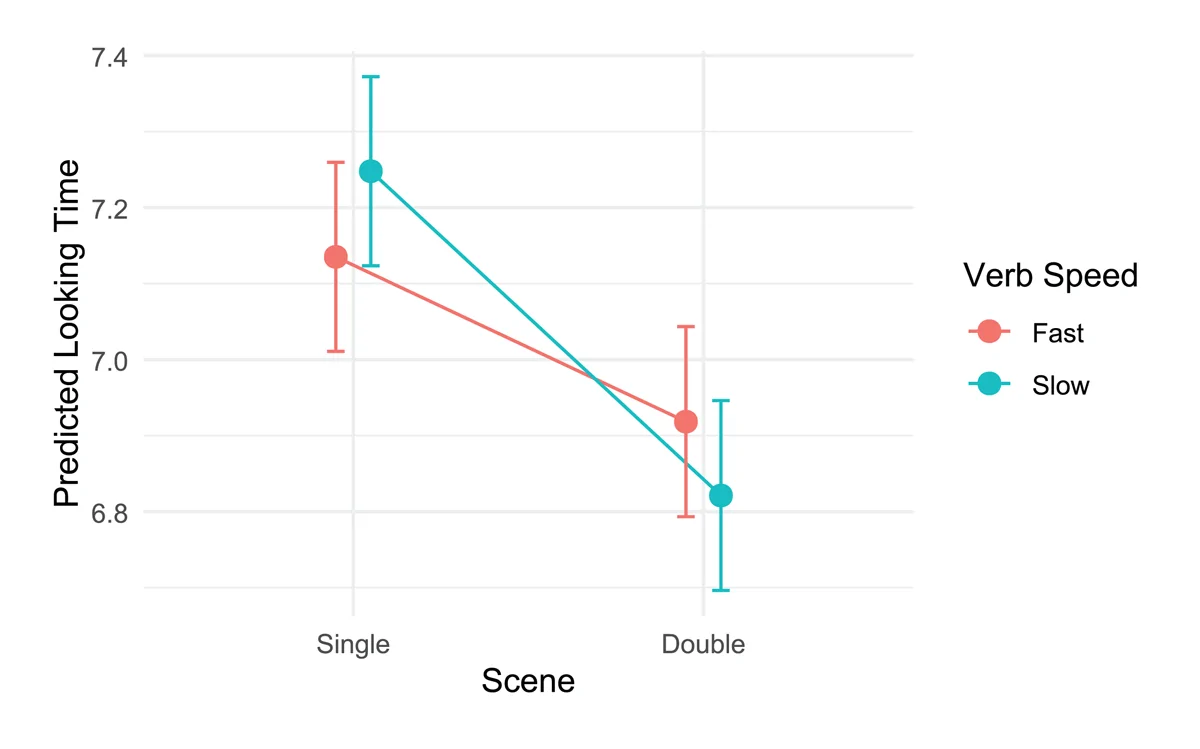
Estimated Marginal Means (EMMs) of the Total Dwell Time on Goal by Verb Speed and Scene. *Note*. EMMs are based on the linear mixed-effects model. Error bars represent 95% confidence intervals.

For looks to the Agent, there were no significant effects. The estimated difference between the fast and slow condition was 0.10 (95% CI [–0.22, 0.22]) in single scenes and 0.01 (95% CI [–0.11, 0.13]) in double scenes. For looks to the Path, there was a main effect of Scene, reflecting the fact that the gaze was distributed between a larger number of objects such that overall looking time of each object decreased and that Path entities depicted in double scenes were shorter than in single scenes (but note the low IA coverage rate for Path as shown in Table 4: out of 320 trials in condition, Path was fixated in 63% and 66% of single trials, and only 19% and 23% of double trials). The estimated difference for Path was –0.08 (95% CI [–0.27, 0.10]) in single scenes and 0.02 (95% CI [–0.25, 0.29]) in double scenes. For looks to Goal, there was a significant interaction between Verb Speed and Scene (χ^2^(1) = 10.03, *p* = 0.002), with shorter looking times on the Goal with fast verbs compared to slow verbs in single scenes, and shorter looking times on the Goal with slow verbs compared to fast verbs in double scenes. The estimated difference for Goal between fast and slow was –0.11 (95% CI [–0.25, 0.03]) in single scenes and 0.10 (95% CI [–0.04, 0.24]) in double scenes, as shown in Figure 7. This indicates that within scene types, the difference between fast and slow sentences is not significant. The interaction therefore shows that the difference between fast and slow sentences differs depending on scene type. Note, however, that in the narrow window analysis the interaction was not significant; see Appendix A.

We again conducted further analyses using Bayes Factors in terms of BF_10_ values (this was not pre-registered, but was decided to be added during data analysis). For looks to the Agent, BF_10_ values provide evidence favouring the models without the main effect of Verb Speed (BF_10_ = 0.18–0.26 across priors of 0.3, 0.4 and 0.5); for the Verb Speed × Scene interaction, the evidence either favoured the models excluding the interaction or was inconclusive (BF_10_ = 0.30–0.45). For looks to the Path, the evidence was in favour of the models excluding the main effect of Verb Speed (BF_10_ = 0.20–0.30) and the interaction (BF_10_ = 0.20–0.33). For looks to the Goal, there was moderate-to-strong evidence favouring the models excluding the main effect of Verb Speed (BF_10_ = 0.15–0.23), but the Verb Speed × Scene interaction showed evidence in favour of the models including the interaction (BF_10_ = 11.56–17.32). However, the within-scene simple effects of Verb Speed were not supported by the data, as calculating BF_10_ values separately for the two scene types showed that evidence is mostly inconclusive for the effect of Verb Speed (BF_10_ = 0.44–0.64 in single scene dataset and BF_10_ = 0.30–0.44 in double scene dataset).

In sum, the results for the total dwell time are inconclusive, with some indications for a significant Verb Speed × Scene interaction, but inconclusive evidence for Verb Speed as a main effect within each scene type. If the tendencies we observed for looks to the Goal in response to slow verbs with single scenes were supported by more conclusive evidence, that would mirror the findings of Speed and Vigliocco (2014) and diverge from Lindsay et al. (2013) even though the structure of the linguistic stimuli (the inclusion of Path phrases in the motion descriptions) followed that of Lindsay et al. (2013).

#### 3.2.3 Time-course Analysis

**Figure 8:**
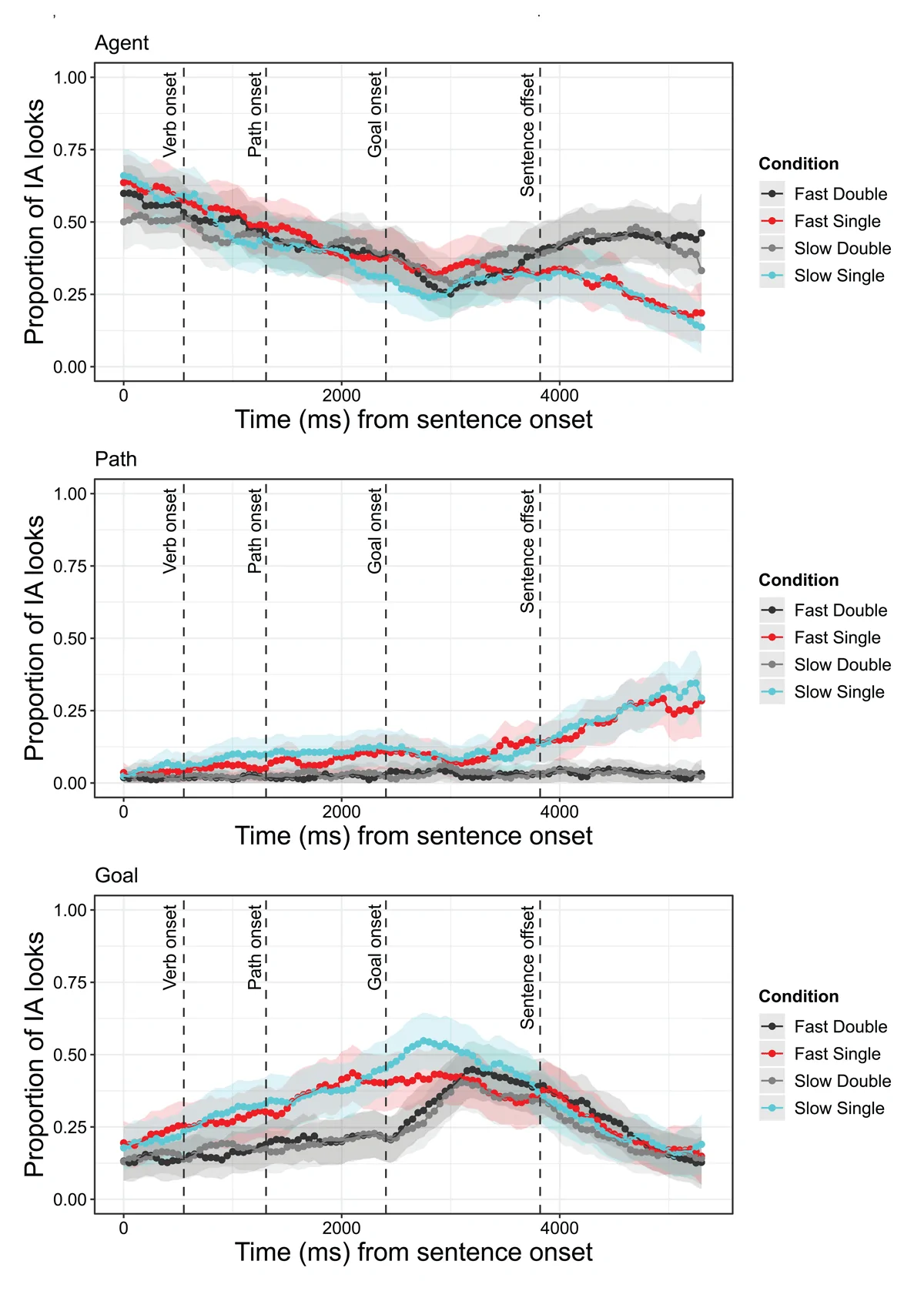
Grand Averages of the Proportions of Looks to the IAs across the Trial Time Starting from Sentence Onset in Experiment 2. *Note*. The mean verb onset was 552 ms (*SD =* 78, range 399–690 ms), Path phrase onset was 1306 ms (*SD =* 135 ms, range 1015–1564 ms), and Goal phrase onset was 2406 ms (*SD* = 152 ms, range 2159–2735 ms). Shaded areas indicate 95% confidence intervals.

The proportions of fixations to one of the three interest areas during the course of sentence listening is highlighted in Figure 8. Regarding Agent and Path, within one scene type (single vs. double), there are no observable differences between the fast and slow condition. Regarding Goal, and in line with the LMER modelling, there is a large difference between the fast and slow condition in single scenes, emerging shortly before the mean onset of the Goal phrase. Whereas fixations towards the Goal entity remain stable with fast verbs, the proportion of fixations increases greatly with slow verbs.

**Figure 9:**
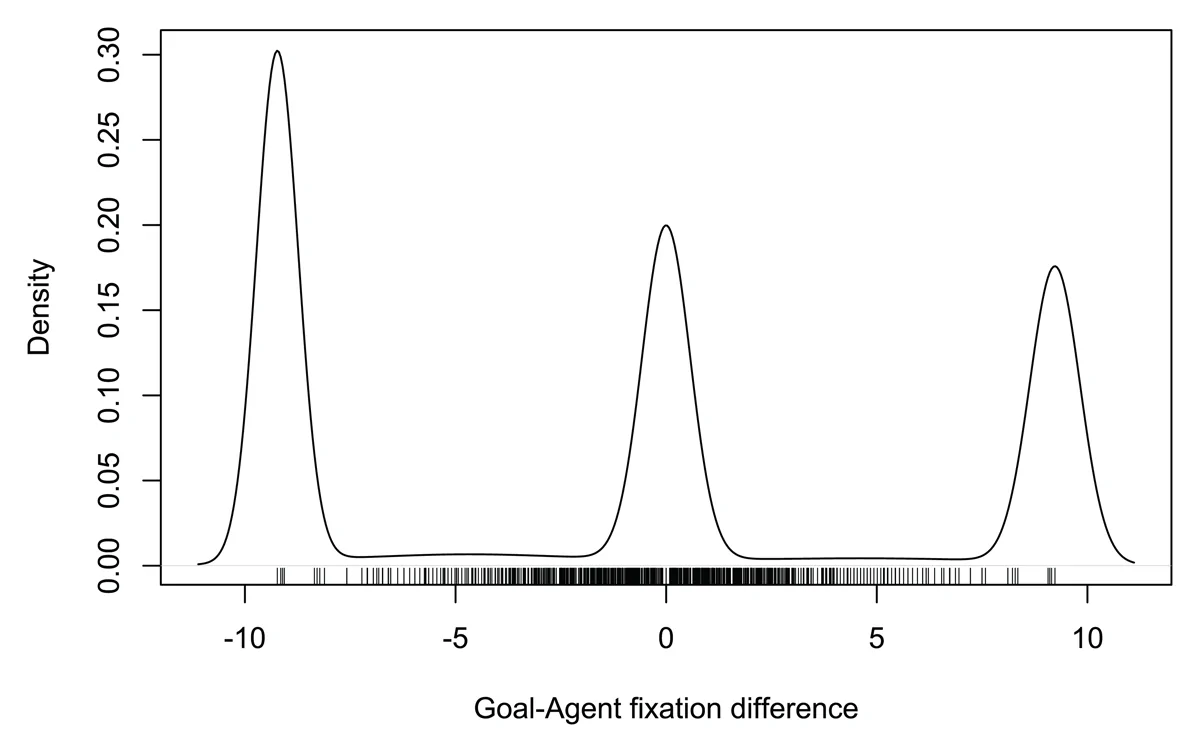
Density Plot of the Variable Goal–Agent Fixation Difference in Experiment 2.

As in Experiment 1, we use GAMM modelling to predict the fixation difference falling on the IAs using the difference between empirical logits of Goal and Agent (see Figure 9). This analysis was not pre-registered. We also use Condition as the interaction term between Verb Speed and Scene as the four-level predictor variable (Fast Single, Slow Single, Fast Double, Slow Double), with Fast Single as the reference level. As non-linear smooths, Time, Verb Duration and Verb Frequency were added, with Time interacting with the four-level Condition. In addition, the model included by-participant and by-item random smooths of Time. The autocorrelation parameter AR1 included in the final model was established as .94.

The GAMM model summary indicates that at sentence onset, single and double scenes are different in Goal-directed fixations (see Table 6). Smooth terms show that attention allocation across time is significantly different from zero.

**Table 6:** Summary of GAMM Results Predicting Goal–Agent Fixation Difference in Experiment 2.


A. PARAMETRIC COEFFICIENTS				

PREDICTOR	ESTIMATE	*SE*	*t*	*p*

(Intercept)	–0.724	0.250	–2.892	.004**

Condition (Fast Double)	–0.929	0.266	–3.488	<.001***

Condition (Slow Double)	–1.050	0.347	–3.022	.003**

Condition (Slow Single)	0.503	0.347	1.448	.148

**B. SMOOTH TERMS**				

**SMOOTH TERM**	***Edf***	***Ref. df***	***F***	***p***

s(Time): Condition (Fast Single)	4.115	5.019	7.383	<.001***

s(Time): Condition (Fast Double)	7.979	8.658	9.020	<.001***

s(Time): Condition (Slow Double)	6.437	7.506	4.426	<.001***

s(Time): Condition (Slow Single)	7.233	8.164	10.438	<.001***

s(Time, Subject) (random effect)	189.889	359.000	1.320	<.001***

s(Time, Item) (random effect)	119.171	284.000	0.801	<.001***

s(Verb Frequency)	1.533	1.681	3.183	.121

s(Verb Duration)	1.596	1.754	0.488	.619


*Note*. Model fit: Adjusted *R*² = .110; Deviance explained = 11.2%; *f*REML = 302,250; Scale estimate = 42.842; *n* = 136,588. *Edf* = estimated degrees of freedom. *Ref. df* = reference degrees of freedom. Significance codes: *** *p* < .001; ** *p* < .01; * *p* < .05.

**Figure 10:**
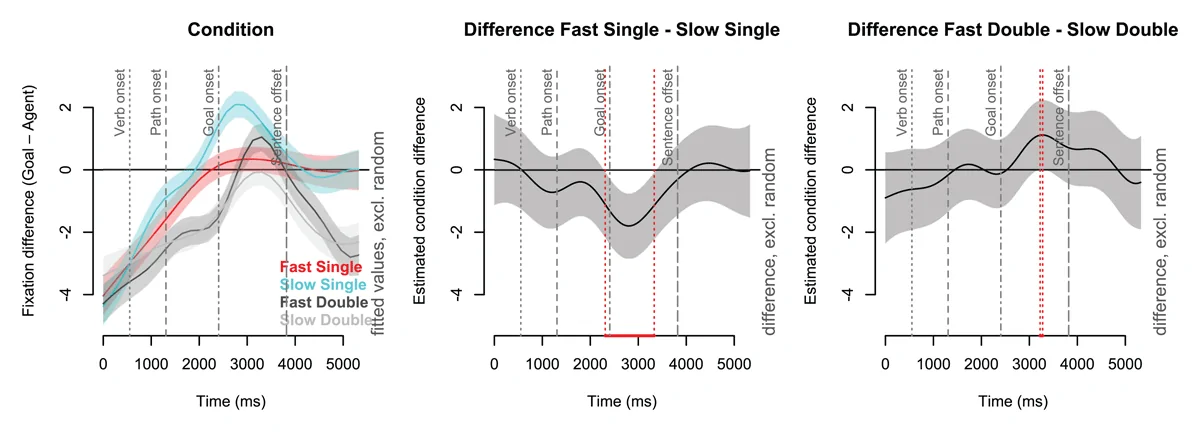
GAMM-estimated Smooths and Condition Differences in Experiment 2. *Note*. The smooths for the effect of Condition on Goal–Agent fixation difference (difference between the empirical logits of Goal and Agent) are on the left panel. GAMM-derived difference curves for the effect of Verb Speed on Goal–Agent fixation difference are shown for single scenes (middle panel) and double scenes (right panel). The x-axis is time in milliseconds, with 0 ms marking the onset of the audio.

The smooth and difference plots in Figure 10 confirm the visual observation made in Figure 8 that fast and slow sentences in single scenes differ significantly, starting at 2311 ms and until 3332 ms, which corresponds roughly to the time the Goal phrase was heard. In this time window of single scenes, there are more looks to the Goal with slow sentences compared to fast sentences. In double scenes, a significant difference between fast and slow sentences is detected around 3250 ms where there is a slight Goal-bias in response to fast sentences. Again, the results are the same with the zero timepoint set to verb onset and when the dependent variable is Goal (see supplementary materials on the OSF).

#### 3.2.3 Summary Experiment 2

In sum, when processing longer sentences (including a Path phrase), a significant interaction implied a pattern where total looking times to the Goal were longer with slow verbs compared to fast verbs in single scenes, but longer looking times to the Goal with fast verbs compared to slow verbs in double scenes. However, the observed differences are small and the exploratory EMM analyses showed that the corresponding within-scene effects were not significant. Bayes Factors, added as an exploratory analysis, suggested that the within-scene evidence was inconclusive, whereas they provided evidence favouring the Verb Speed × Scene interaction. Thus, the results should be treated with caution. When looking at the time course of fixations, a similar trend occurred, which appeared more pronounced in single scenes where the Goal tended to be fixated on more with slow, rather than fast sentences, in the postverbal time of the Goal phrase.

## 4. General Discussion

In two conceptual replication experiments, we examined the mental simulation of speed by measuring whether speed-induced language affects online processing in Estonian. We used the visual world paradigm and recorded eye movements while participants heard sentences describing fast and slow motion, following the design of Speed and Vigliocco (2014) in Experiment 1 as closely as possible (apart from language) and extending to more closely match the sentence structure used in Lindsay et al. (2013) in Experiment 2 (apart from tense choice).

We found that when sentences were shorter (comprising a mover noun, motion verb expressing either fast or slow motion, and destination phrase), no significant differences in eye-movement patterns emerged, contrary to Speed and Vigliocco (2014). However, when sentences were longer (i.e., comprising a mover noun, motion verb, path and destination, as in Lindsay et al. (2013)), the interaction between scene type and verb speed became significant, suggesting that the direction of the difference between fast and slow verbs differed depending on the scene type. Specifically, when no distractors were present in the scene, the Goal received shorter looking times in response to sentences of fast motion than slow motion. In contrast, when a distractor was present, the opposite pattern occurred in that the Goal was looked at longer when the verb referred to fast motion relative to verbs referring to slow verbs. Nevertheless, while the interaction was significant, the exploratory simple effects analyses of verb speed were not and thus, the results should be interpreted with caution. The tendencies we observed for single scenes are in line with Speed and Vigliocco (2014) but opposite to Lindsay et al. (2013). In addition, contrary to the original studies where Goal attraction emerging over time was stronger for fast than for slow sentences (Lindsay et al., 2013; Speed & Vigliocco, 2014), the current time-course analysis of Experiment 2 indicated that the Goal was looked at earlier while listening to sentences expressing slow motion, and this was also significant in the exploratory GAMM modelling. Thus, the study reveals mixed findings. In addition, the Path was infrequently fixated, ranging from 13% to 36% in Experiment 1 and from 19% to 66% in Experiment 2 across conditions, hindering any conclusions for this IA, apart from the observation that explicitly mentioning the Path in Experiment 2 increased attention to this entity in the scenes.

The fact that significant results were found in Experiment 2 (longer sentences) but not in Experiment 1 (shorter sentences) may relate to the notion that mental simulation requires time to unfold, as also suggested by several studies (e.g., Hoeben Mannaert et al., 2019; Pan et al., 2024; Speed & Vigliocco, 2014). For example, Speed and Vigliocco (2014) found effects of verb speed in sentences spoken slowly but not sentences spoken quickly. The likelihood of participants fixating on interest areas increases with longer sentences (i.e., longer trials) and longer duration may also aid comprehension. In Experiment 2, the number of participants that had to be excluded due to them not fixating on IAs or due to low accuracy on comprehension questions was considerably smaller than in Experiment 1. Alternatively, rather than sentence length, the semantic richness of Experiment 2 might have influenced the results. Adding path information to the sentence increases event detail which could result in more in-depth processing, which, in turn, can be a prerequisite for mental simulation (see also Barsalou et al., 2008; Connell, 2019; Vukovic et al., 2017).

Individual and contextual differences may relate to the study’s mixed findings. In this study, participants had to simply listen to the sentences and answer a subsequent comprehension question. In this setup, different strategies are available for completing the task, which do not necessarily require mental simulation. For example, it is possible to answer the comprehension questions based solely on auditory information, without relying on the visual input. Naturally, this would affect eye-movement patterns, as participants might simply stare at the centre of the screen or focus on visual features that capture attention. Four participants who strongly relied on this strategy were excluded from Experiment 1. Of the remaining 40 participants in Experiment 1, six had missing data for 10 to 17% of the IAs (i.e., Agent or Goal), and 23 had missing data for 1 to 10% of the IAs. Only 10 participants had fixations recorded on IAs in all trials. In Experiment 2, no participants were excluded due to missing data on Agent or Goal IAs. The highest proportion of missing data was 7%, with the rest of the 23 participants showing between 1 to 5% missing data. Seventeen participants had fixations on both Agent and Goal in all trials. In other words, and particularly with shorter sentences, it is conceivable that task-related factors, to which mental simulation is known to be sensitive (e.g., Von Sobbe et al., 2021), or individual comprehension strategies which may also vary in the context of mental simulation (Ibáñez et al., 2023; see also Winter et al., 2022), influenced the observed eye-movement patterns. Using the visual world paradigm with spoken sentences and no specific task beyond comprehension may therefore not always effectively capture mental simulation. Alternatively, more time, richer linguistic context, or goal-directed language comprehension (e.g., listening to instructions) may be required for mental simulation to take place.

It is also necessary to consider whether the eye-movement differences we observed between sentences with fast and slow verbs in Experiment 2 reflect processes other than mental simulation. For example, eye-movements are sensitive to factors such as visual saliency and semantic congruency (Underwood & Foulsham, 2006), or predictability of referents (e.g., Altmann & Kamide, 1999). It seems unlikely that such factors explain the patterns observed here however because sentences and corresponding visual scenes were matched across fast and slow versions of the sentences. Nevertheless, language-mediated processes other than mental simulation might have occurred that influenced the looking behaviour (e.g., based on verb collocations or non-speed-related lexical associations). It is also possible that a phenomenon known as central bias (Fehd & Seiffert, 2010) took place, where participants strategically focus on one point rather than swich between relevant targets.

This study also addresses one aspect that has received relatively little attention in the literature: the language used to test mental simulation. One might predict that if mental simulation occurs, it is somewhat universal – at least as a cognitive mechanism – since there is currently no strong evidence supporting the idea that language alone drives cognitive processes (i.e., strong linguistic relativity). This would imply that mental simulation should occur regardless of the specific language used. However, language-related factors still play a role, as structural properties and the situational or cultural information embedded in a given language can modulate mental simulation. At the same time, research on mental simulation (or embodiment more broadly) is heavily biased toward Indo-European languages, as also demonstrated by recent large-scale replication studies (Morey et al., 2022; Winter et al., 2022). Consequently, relatively little is known about how mental simulation effects manifest in non-Indo-European languages.

Regarding the current study, although the Estonian language allows the same word order as English (subject–verb–adverbial), there are nevertheless crucial differences between the two languages, which in turn influence the precise timing of when participants could hear key content words. One main difference is that Estonian does not have articles, meaning that all noun or adpositional phrases are typically one word shorter than in English in this respect. Secondly, to express spatial information, Estonian uses case endings or adpositions, mainly postpositions. In the current experiment, Goal phrases consisted of a noun followed by a postposition *juurde* ‘to’ denoting the Goal (e.g., *puu juurde* [tree.gen to.postp] ‘to the tree’). Whether the placement of the goal marker (before the noun in English or after the noun in Estonian) affected the results cannot be determined based on the current data or existing knowledge in the field. Another factor is that of tense. The linguistic stimuli in Estonian were in the past simple form to mimic the study by Speed and Vigliocco (2014). This ultimately means a deviation from the Lindsay et al.’s (2013) study, as in their experiment, the sentences were in the future tense. Estonian has no grammatical future tense, and the present tense is used instead. It cannot be ruled out that tense differences between the two original studies and the chosen tense form in Estonian also impact the results, as tense and aspect constructions have been shown to influence mental simulation (e.g., Bergen & Wheeler, 2010). This highlights the need to expand mental simulation research to lesser-studied languages, while also considering nuanced grammatical information in order to better account for linguistic diversity.

Taken together, the data provide inconsistent support for mental simulation of speed during sentence comprehension. Given the mixed findings of the current study and the contrasting pattern of results with previous studies, it is difficult to make conclusions about the extent to which mental simulation results from speed-induced language and/or affects looking behaviours. Mental simulation of speed may be more sensitive to context than mental simulation of other information, such as colour (e.g., Connell, 2007; de Koning, Wassenburg, Bos, & van der Schoot, 2017; Hoeben Mannaert et al., 2017) due to the complexity involved in integrating spatial and temporal information (Speed & Vigliocco, 2014). In addition, mental simulation can be sensitive to participant variability in processing strategies, which the current study failed to account for. In general, more research using diverse tools and designs, and incorporating a wider range of languages, is needed to better understand mental simulation – and the simulation of speed language in particular – and the role of language in human–world interaction.

## Data Availability

Eye-tracking data and R code are available on OSF 10.17605/OSF.IO/QTX8W

## Footnotes

Two additional hypotheses were pre-registered for total dwell time: dwell time on the Agent and Goal in scenes with a distractor will be shorter than in scenes without a distractor. We do not address these hypotheses here as they have little relevance to our main research question.

## Appendix A

### LMER model outputs

Model output for the total dwell time in Experiment 1 (wide time window)

**Table:**

*PREDICTORS*	AGENT				PATH				GOAL			
		
	*EST.*	*SE*	*t*	*p*	*EST.*	*SE*	*t*	*p*	*EST.*	*SE*	*t*	*p*

(Intercept)	5.96	0.42	14.13	**<0.001**	4.91	047	10.38	**<0.001**	7.55	0.38	20.10	**<0.001**

Verb Speed [Slow]	0.06	0.07	0.83	0.406	–0.05	0.08	–0.57	0.569	–0.07	0.07	–1.06	0.287

Scene [Double]	0.01	0.04	0.21	0.835	–0.58	0.11	–5.37	**<0.001**	–0.43	0.05	–9.15	**<0.001**

Verb Frequency	0.11	0.04	2.68	**0.008**	0.09	0.04	2.15	**0.032**	–0.03	0.04	–0.72	0.474

Verb Duration	0.00	0.00	1.37	0.171	0.00	0.00	1.56	0.119	–0.00	0.00	–1.25	0.213

Verb Speed [Slow] × Scene [Double]	–0.05	0.06	—0.83	0.404	0.24	0.15	1.57	0.118	0.05	0.07	0.79	0.427

**Random Effects**												

σ^2^	0.31				0.38				0.33			

τ_00_	0.03_participant_				0.01_participant_				0.05_participant_			

	0.02_item_				0.00_item_				0.02_item_			

ICC	0.14								0.16			

N	40_participant_				40_participant_				40_participant_			

	32_item_				32_item_				32_item_			

Observations	1257				335				1195			

Marginal R^2^/Conditional R^2^	0.026/0.165				0.126/NA				0.101/0.245			

Model output for the total dwell time in Experiment 1 (narrow time window)

**Table:**

PREDICTORS	AGENT				PATH				GOAL			
		
	*EST.*	*SE*	*t*	*p*	*EST.*	*SE*	*t*	*p*	*EST.*	*SE*	*t*	*p*

(Intercept)	5.63	0.40	14.18	**<0.001**	4.90	0.47	10.52	**<0.001**	7.43	0.34	22.02	**<0.001**

Verb Speed [Slow]	0.05	0.07	0.75	0.451	–0.07	0.08	–0.94	0346	–0.10	0.06	–1.56	0.119

Scene [Double]	0.08	0.05	1.49	0.136	–0.59	0.11	–5.54	**<0.001**	–0.41	0.05	–8.34	**<0.001**

Verb Frequency	0.08	0.04	2.04	**0.042**	0.09	0.04	2.29	**0.023**	–0.04	0.03	–1.28	0.202

Verb Duration	0.00	0.00	2.01	**0.045**	0.00	0.00	1.57	0.118	–0.00	0.00	–1.05	0.293

Verb Speed [Slow] × Scene [Double]	–0.04	0.07	—0.52	0.606	0.27	0.15	1.79	0.074	0.09	0.07	1.24	0.215

**Random Effects**												

σ2	0.39				0.35				035			

τ00	0.04participant				0.01participant				0.05participant			

	0.02item				0.00item				0.01item			

ICC	0.13								0.14			

N	40participant				40participant				40participant			

	32item				32item				32item			

Observations	1158				328				1149			

Marginal R2/Conditional R2	0.019/0.144				0.134/NA				0.084/0.213			

Model output for the total dwell time in Experiment 2 (wide time window)

**Table:**

*PREDICTORS*	AGENT				PATH				GOAL			
		
	*EST.*	*SE*	*t*	*p*	*EST.*	*SE*	*t*	*p*	*EST.*	*SE*	*t*	*p*

(Intercept)	6.88	0.32	21.21	**<0.001**	5.80	0.55	10.61	**<0.001**	7.10	0.39	18.39	**<0.001**

Verb Speed [Slow]	–0.10	0.06	–1.70	0.089	0.08	0.09	0.95	0.344	0.11	0.07	1.67	0.095

Scene [Double]	0.07	0.04	1.78	0.075	–0.65	0.10	–6.31	**<0.001**	–0.22	0.05	–4.64	**<0.001**

Verb Frequency	0.07	0.03	2.19	**0.029**	0.03	0.05	0.63	0.529	–0.03	0.04	–0.73	0.463

Verb Duration	0.00	0.00	0.61	0.539	0.00	0.00	0.69	0.490	0.00	0.00	0.72	0.472

Verb Speed [Slow] × Scene [Double]	0.09	0.06	1.55	0.121	–0.10	0.14	–0.72	0.473	–0.21	0.07	–3.17	**0.002**

**Random Effects**												

σ^2^	0.28				0.46				0.34			

τ_00_	0.03_participant_				0.09_participant_				0.06_participant_			

	0.01_item_				0.02_item_				0.02_item_			

ICC	0.13				0.20				0.18			

N	40_participant_				40_participant_				40_participant_			

	32_item_				32_item_				32_item_			

Observations	1267				548				1244			

Marginal R^2^/Conditional R^2^	0.025/0.153				0.142/0.311				0.067/0.237			

Model output for the total dwell time in Experiment 2 (narrow time window)

**Table:**

PREDICTORS	AGENT				PATH				GOAL			
		
	*EST.*	*SE*	*t*	*p*	*EST.*	*SE*	*t*	*p*	*EST.*	*SE*	*t*	*p*

(Intercept)	6.70	0.36	18.61	**<0.001**	5.76	0.48	11.93	**<0.001**	6.96	0.36	19.13	**<0.001**

Verb Speed [Slow]	–0.08	0.07	–1.24	0.214	0.06	0.08	0.81	0.418	0.14	0.07	2.17	**0.030**

Scene [Double]	0.10	0.05	2.03	**0.042**	–0.65	0.11	–6.09	**<0.001**	–0.20	0.05	–4.17	**<0.001**

Verb Frequency	0.05	0.04	1.34	0.180	0.03	0.04	0.68	0.495	–0.02	0.04	–0.70	0.485

Verb Duration	0.00	0.00	0.63	0.527	0.00	0.00	0.90	0370	0.00	0.00	0.90	0.370

Verb Speed [Slow] × Scene [Double]	0.10	0,07	1.42	0.156	–0.08	0.14	–0.53	0.597	–0.26	0.07	–3.78	**<0.001**

**Random Effects**												

σ2	0.36				0.48				0.35			

τ00	0.03_participant_				0.09_participant_				0.06_participant_			

	0.01_item_				0.01_item_				0.01_item_			

ICC	0.11				0.17				0.18			

N	40_participant_				40_participant_				40_participant_			

	32_item_				32_item_				32_item_			

Observations	1206				533				1214			

Marginal R2/Conditional R2	0.019/0.129				0.134/0.278				0.071/0.241			
